# Effects of Zinc Oxide Nanoparticles on Model Systems of the Intestinal Barrier

**DOI:** 10.3390/toxics10020049

**Published:** 2022-01-21

**Authors:** Anna Mittag, Patricia Owesny, Christian Hoera, Alexander Kämpfe, Michael Glei

**Affiliations:** 1 Department of Applied Nutritional Toxicology, Institute of Nutritional Sciences, Friedrich Schiller University Jena, Dornburger Straße 24, 07743 Jena, Germany; patricia.owesny@dife.de (P.O.); michael.glei@uni-jena.de (M.G.); 2 Swimming and Bathing Pool Water, Chemical Analytics, German Environment Agency, Heinrich-Heine-Straße 12, 08645 Bad Elster, Germany; christian.hoera@uba.de (C.H.); alexander.kaempfe@uba.de (A.K.)

**Keywords:** barrier integrity, Caco-2, coculture, HT29-MTX, nanoparticles, toxicity, Transwell, zinc oxide

## Abstract

Zinc oxide nanoparticles (ZnO NP) are often used in the food sector, among others, because of their advantageous properties. As part of the human food chain, they are inevitably taken up orally. The debate on the toxicity of orally ingested ZnO NP continues due to incomplete data. Therefore, the aim of our study was to examine the effects of two differently sized ZnO NP (<50 nm and <100 nm primary particle size; 123–614 µmol/L) on two model systems of the intestinal barrier. Differentiated Caco-2 enterocytes were grown on Transwell inserts in monoculture and also in coculture with the mucus-producing goblet cell line HT29-MTX. Although no comprehensive mucus layer was detectable in the coculture, cellular zinc uptake was clearly lower after a 24-h treatment with ZnO NP than in monocultured cells. ZnO NP showed no influence on the permeability, metabolic activity, cytoskeleton and cell nuclei. The transepithelial electrical resistance was significantly increased in the coculture model after treatment with ≥307 µmol/L ZnO NP. Only small zinc amounts (0.07–0.65 µg/mL) reached the basolateral area. Our results reveal that the cells of an intact intestinal barrier interact with ZnO NP but do not suffer serious damage.

## 1. Introduction

Nanoparticles (NP) have become an indispensable part of our daily life. NP of metal oxides, such as zinc oxide (ZnO), are among the most produced NP [1]. ZnO NP offer distinguished properties, such as photocatalytic, bio-imaging, anti-bacterial and anti-inflammatory abilities, with a higher aspect ratio and a large surface-area-to-volume ratio compared with that of the bulk material [2]. This provides a wide application range. For example, ZnO NP are used in sunscreen lotions, transparent transistors, solar cells, memory devices, cosmetics, catalysis, medicine and photoconductive materials [3]. They improve wound healing, cancer therapies and diabetes treatments [2,4]. Furthermore, ZnO NP are used in the food sector as a food additive, where they serve as a dietary source of zinc, or as a preservative in food packaging, where they act as an anti-microbial agent to protect food from microbial contamination [5].

Because of their diverse applications, large amounts of ZnO NP are released into the environment [6]. Due to their high surface activity and adsorption properties, they may cause adverse biological effects. The potential risks of ZnO NP to ecological and biological treatment systems have caused great concern in recent years, and increasing attention has been given to biotoxicity [6,7,8].

The gastrointestinal tract is one of the main routes of ZnO NP uptake into the body. They can be absorbed directly upon ingestion in the human intestinal tract and be translocated into the circulation [7]. It is already known that compounds of zinc are possible toxicants for the ecological system [5], and the toxicity of ZnO NP in food products has been a subject of research for many years. Previous studies on the toxic effects of orally administered ZnO NP on cells and tissues produced contradictory results. While some observed damage to intestinal epithelial barrier integrity [9], pathological gut changes [7], immunotoxicity in thymus and spleen [10], harmful effects in brain tissue [11] or neurotoxic potential [12,13], others indicated positive effects, such as improved antioxidative capacities and less intestinal injury after ZnO NP supplementation [14], increased immune response and antioxidant defence [15] and an improved antioxidative status [16]. Additionally, the impact of the primary particle size of ZnO NP on toxicity is still ambiguous [17,18,19,20]. The data available thus far on the effects of orally ingested ZnO NP are insufficient [21,22,23]. To change this situation, appropriate in vitro studies considering the complex structure of the gastrointestinal tract could be helpful.

The intestinal epithelium consists of various differentiated cell types, especially absorptive enterocytes and goblet cells, which proceed with unique and specialized functions [24]. The Caco-2 cell line is an accepted in vitro model to mimic the intestinal epithelium of the small intestine since differentiated Caco-2 cells are functionally and structurally like enterocytes in vivo [25]. HT29-MTX cells are mucus-secreting cells, which are used for mimicking goblet cells in vitro [26]. Both cell lines can be grown as a coculture with barrier properties that are similar to the human intestine [27].

In this study, differentiated Caco-2 cells were used as a monoculture and additionally as coculture with the HT29-MTX cell line. In an approximation of physiological conditions, cells were grown on Transwell inserts, which allow separation into apical and basolateral compartments. Following exposure to ZnO NP of two different sizes, barrier integrity, permeability, cellular zinc uptake and permeation through the monolayer were investigated.

## 2. Materials and Methods

### 2.1. Preparation and Characterization of ZnO NP Dispersions

The preparation of ZnO NP dispersions has already been described in detail [28] and was performed according to DeLoid and Cohen [29] to ensure a comparable quality of the used particle dispersions. Before each experiment, fresh 1 mg/mL (12,990 µmol/L) ZnO NP stock dispersions were made by mixing ZnO nanopowder (#677450 and #544906 from Sigma Chemical Corp., Taufkirchen, Germany) with Millipore filtered water and added to a glass rosette cell (RZ2; Bandelin electronic GmbH & Co. KG, Berlin, Germany), which was submerged in an ice bath to avoid heat generation. An ultrasonic homogenizer (Sonopuls HD 2070; Bandelin electronic GmbH & Co. KG, Berlin, Germany) was immersed 1 cm deep in the ZnO NP stock dispersions, which were then ultrasonicated for 1 h (critical sonication energy: 720 J/mL). Subsequently, the dispersions were mixed with cell culture medium. Using the Zetasizer Nano ZS (Malvern Panalytical GmbH, Kassel, Germany), the mean hydrodynamic diameter and polydispersity index of the ZnO NP dispersions were measured via dynamic light scattering, and the zeta potential was measured via electrophoretic light scattering. Measurements of the specific surface area, solubility and stability in Millipore filtered water and cell culture medium, as well as investigations of primary particle size and shape using transmission electron microscopy, had already been carried out [30].

### 2.2. Cell Culture and NP Exposure

Two different, functionally complementary colon adenocarcinoma cell lines were used as the model system for the intestinal epithelium. Caco-2 cells exhibit morphological and functional resemblances to small intestinal epithelial cells through differentiation. They form a characteristic polarization with a brush border and microvilli, as well as cell-cell contacts on the apical side, and they produce enterocyte-specific enzymes. Therefore, they are a suitable in vitro model system for studying intestinal barrier functions [31]. The Caco-2 cell line was purchased from ATCC (LGC Standards GmbH, Wesel, Germany). For cell culture experiments, passages 22–40 were used. In contrast, HT29 cells form an adherent monolayer of unpolarized and undifferentiated epithelial-like cells. In modified cell culture medium, they can differentiate and acquire other morphological and functional characteristics. The addition of methotrexate (MTX) induces differentiation into mucus-producing HT29-MTX cells, which are similar to goblet cells in the gastrointestinal tract. They represent an appropriate model for investigating the transport and uptake of various substances through the epithelial barrier [31]. The HT29-MTX cell line was a kind gift from Ms. N. Jablonowski of the research group Microbial Pathogenicity Mechanisms (Leibniz Institute for Natural Product Research and Infection Biology, Hans Knöll Institute, Jena, Germany). Passages between 4 and 22 were used for the experiments. Both cell lines have been verified by short tandem repeats profiling.

Caco-2 and HT29-MTX cells were cultured in Dulbecco’s Modified Eagle Medium, which was complemented with 10% fetal bovine serum (FBS), 1% non-essential amino acids and 1% penicillin/streptomycin (media and additives from PAN-Biotech GmbH, Aidenbach, Germany) in an incubator (37 °C, 95% humidity, 5% CO_2_; Thermo Fisher Scientific Inc., Waltham, MA, USA). The cells were periodically examined for mycoplasma contamination. For the experiments, cells were seeded on ThinCert™ cell culture inserts in 12-well plates (3 µm pore size; Greiner Bio-One International GmbH, Frickenhausen, Germany). The monoculture consisted of Caco-2 cells, and the coculture also contained HT29-MTX cells (ratio 3:1; altogether 33,000 cells per well). To obtain a differentiated and stable mono- and coculture system, the cells were cultivated for 21 days, and the cell culture medium was changed every 2–3 days.

After differentiation, the cells were treated with ZnO NP dispersed in cell culture medium at concentrations of 123–614 µmol/L. The chosen ZnO NP concentrations were in accordance with Sohal and DeLoid [32]. They calculated realistic in vitro doses at ranges comparable to in vivo human doses using in vitro dosimetry models, which also included available human uptake data and knowledge of gastrointestinal tract physiology. The estimated realistic in vitro concentration of ZnO NP (about 246 µmol/L) is analogous with that of TiO_2_ since there is no information about the daily intake of ZnO NP. However, both NP are used in similar areas and quantities. A range around this concentration was chosen for our studies. Zinc chloride (ZnCl_2_) was used in equimolar concentrations and served as a source of free zinc ions. Cell culture medium without additives was used as an untreated control. The solvent control contained 5% Millipore filtered water to consider potential effects caused by the solvent. The positive control consisted of 0.1% Triton X-100 (Merck KGaA, Darmstadt, Germany) and 10 mM ethylene glycol-bis(2-aminoethlyether)-N,N,N′,N′-tetraacetic acid (EGTA; Carl Roth GmbH & Co. KG, Karlsruhe, Germany) on the apical side and 10 mM EGTA on the basolateral side.

### 2.3. Alcian Blue Staining

To confirm mucus production in HT29-MTX cells, alcian blue staining was conducted. The cationic polyvalent dye alcian blue binds acidic polysaccharides, such as glycosaminoglycans, mucopolysaccharides and sialylated glycocalyx [33]. For this, Caco-2 and HT29-MTX cells were cultivated both as monoculture and as coculture in 6-well plates for three weeks to receive differentiated cells. The cell culture medium was replaced every 2–3 days. Subsequently, the cell culture medium was removed, and the cells were fixed with 4% paraformaldehyde (Sigma Chemical Corp., Taufkirchen, Germany) for 20 min at room temperature. The cells were washed with 3% acetic acid to lower the pH. Alcian blue (10 mg/mL, pH 2.5; Merck KGaA, Darmstadt, Germany) was then added, and the cells were stained for 15 min at room temperature. The cells were washed again with 3% acetic acid and covered with phosphate-buffered saline (PBS). The evaluation was carried out microscopically (ZEISS Axio Observer 01; Carl Zeiss AG, Oberkochen, Germany).

### 2.4. MTT Assay

To investigate cellular metabolic activity, the colorimetric MTT (3-(4,5-dimethylthiazol-2-yl)-2,5-diphenyl tetrazolium bromide) assay was used. The cells were washed with PBS after a 24-h treatment with ZnO NP. The cells were incubated with 0.5 mg/mL MTT (stock solution: 50 mg MTT powder (Merck KGaA, Darmstadt, Germany) in 10 mL PBS; working solution diluted with cell culture medium; 500 µL apical, 1000 µL basolateral) for 2 h at 37 °C. After removal of the cell culture medium, dimethyl sulfoxide (Merck KGaA, Darmstadt, Germany; 500 µL apical, 1000 µL basolateral) was added to solubilize the resulting formazan. The cells were placed on an orbital shaker for 10 min. Afterward, the apical and basolateral solutions were mixed well and pipetted from the 12-well plate onto a transparent 96-well plate in triplicate. The absorbance was measured with a microplate reader at 570 nm (reference wavelength: 630 nm; Synergy 2; BioTek Instruments, Inc., Bad Friedrichshall, Germany).

### 2.5. Barrier Integrity Measurements

Three different assays were used to investigate the influence of ZnO NP on monolayer barrier integrity. The transepithelial electrical resistance (TEER) was quantified via chopstick electrodes and an epithelial Volt/Ohm meter (EVOM2; World Precision Instruments GmbH, Friedberg, Germany). An increasing TEER indicates potent intercellular adhesion complexes and cell density [34]. The ohmic resistance was measured both before and after 24 h of incubation with ZnO NP to obtain relative TEER values.

Additionally, changes in monolayer permeability were determined using fluorescein isothiocyanate (FITC)-dextran (average molecular weight 10,000 Da; Sigma Chemical Corp., Taufkirchen, Germany). FITC-based dyes will be transported transcellularly through the monolayer depending on its permeability and can be quantified photometrically. After 24 h of incubation with ZnO NP, the cell culture medium was removed, and phenol red-free medium (PAN-Biotech GmbH, Aidenbach, Germany) was added to the apical and basolateral compartments for 10 min to avoid interference of phenol red with FITC-dextran. Subsequently, the medium was removed again, and 500 µL 1 mg/mL FITC-dextran and 1000 µL phenol red-free medium was added to the apical and basolateral compartments, respectively. Cells were incubated for 30 min at 37 °C on an orbital shaker. Transwell inserts were placed in another 12-well plate with 1000 µL phenol red-free medium in the basolateral compartment for a further 30 min. This step was then repeated, and cells were incubated for a further 5 h. The basolateral medium and the standard series of FITC-Dextran (0.001–1000 µg/mL) were pipetted in triplicate onto a black 96-well plate, and the fluorescence was measured with a microplate reader (excitation: 485 nm, emission: 528 nm; Synergy 2; BioTek Instruments, Inc., Bad Friedrichshall, Germany).

To examine the influence of ZnO NP on the cytoskeleton and cell nucleus, cells were stained with phalloidin and 4’,6’-diamidino-2-phenylindole (DAPI). The bicyclic peptide phalloidin binds to actin filaments, which are a major constituent of the cytoskeleton [35]. DAPI is a DNA-specific fluorochrome, which is used to stain cell nuclei [36]. After 24 h of ZnO NP treatment, cells were washed twice with PBS. They were fixed with 4% paraformaldehyde for 20 min at room temperature and washed again with PBS. Subsequently, cells were permeabilized with 0.1% Triton-X100 for 10 min at room temperature. After two washes with PBS, staining solution containing 0.1 µg/mL DAPI (Sigma Chemical Corp., Taufkirchen, Germany) and 0.1% phalloidin iFlour 488 reagent (Abcam plc., Berlin, Germany) was added, and cells were incubated for 1 h. Finally, the Transwell insert membranes were excised and transferred onto a slide for microscopic evaluation using a laser scanning microscope (LSM 780; Carl Zeiss AG, Oberkochen, Germany).

### 2.6. Quantification of Zinc

Cellular zinc uptake and zinc permeation through the monolayer were investigated using inductively coupled plasma mass spectrometry (ICP-MS). For the first examination, cells were washed twice with PBS after 24 h of ZnO NP exposure. Trypsin/EDTA was added for 5 min at 37 °C. DMEM with 10% FBS was added, and the membrane was rinsed until the cells detached. Detached cells were centrifuged for 5 min at 600× *g*, and the supernatants were removed. Cells were washed with PBS, and cell viability and number were determined using the CASY TT (OLS OMNI Life Science GmbH & Co. KG, Bremen, Germany). The cell suspension was digested overnight with 65% extra pure nitric acid (Suprapur; Merck KGaA, Darmstadt, Germany). To support the digestion, all samples were placed in a sonication bath for 1 h at 50 °C. They were diluted with Millipore filtered water to 2% nitric acid, and the ionic zinc content was measured using ICP-MS (iCAP™ RQ from Thermo Fisher Scientific Inc., Waltham, MA, USA, equipped with the 4DX prepFAST autosampler from ESI Elemental Service & Instruments GmbH, Mainz, Germany) by determining the mass signal *m*/*z* = 66 (corresponds to the zinc isotope with a mass of 66 amu). Rhodium (2 ppb in 2% nitric acid) was used as an internal standard, which was continuously added online to the samples and quantified simultaneously. Three quality check samples with different concentrations along the measurement range were included and measured regularly after about 20 samples. The minimum number, as well as the acceptance criteria for evaluating the quality check samples, were in accordance with the Food and Drug Administration guidelines [37]. The limit of quantification was determined according to DIN 32,645 [38] as 0.3 ± 0.2 µg/L. The measurement uncertainty was calculated according to DIN ISO 11352:2013-03 [39] with four certified reference materials as 7.9%. Data processing was conducted with a program developed from Chemical Analytics (German Environment Agency, Bad Elster, Germany) for quality management, as well as Qtegra™ (Thermo Fisher Scientific Inc., Waltham, MA, USA).

To investigate monolayer permeation, the incubation medium was collected before the treatment and afterward from the apical and basolateral Transwell compartments. The ionic zinc content was measured using ICP-MS as described above. The limit of quantification was 9.0 ± 2.2 µg/L.

### 2.7. Statistical Analysis

Each experimental procedure was repeated independently at least three times. Figures were created using GraphPad Prism 5 (Version 5.01; GraphPad Software, San Diego, CA, USA; 2021). The results shown in the figures are mean values with standard deviations. Data processing was performed by IBM SPSS Statistics (Version 26; IBM Deutschland GmbH, Ehningen, Deutschland; 2021). One-way analysis of variance with the Ryan–Einot–Gabriel–Welsh post hoc test or unpaired *t*-tests were performed to identify significant differences (* *p*  ≤  0.05).

## 3. Results

### 3.1. Characterization of ZnO NP

The physicochemical properties of both ZnO NP used are summarized in Table 1. In cell culture medium (pH value approximately 7.4), dispersed ZnO NP < 50 nm had a hydrodynamic diameter of 162.5 ± 12.0 nm, smaller than that of ZnO NP < 100 nm, which was 219.4 ± 11.3 nm. The polydispersity index was 0.3 ± 0.1 for both ZnO NP. They were slightly negatively charged and had a zeta potential around −9 mV.

### 3.2. Alcian Blue Staining

**Figure 1:**
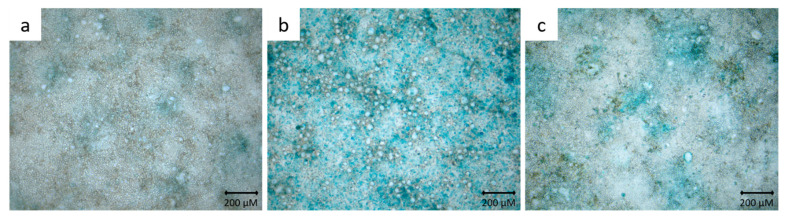
Representative microscopic images of Caco-2 cells (**a**), HT29-MTX cells (**b**) and Caco-2/HT29-MTX coculture cells (**c**) stained with alcian blue.

To demonstrate mucus production in HT29-MTX cells, mono- and cocultured cells were stained with alcian blue. Only single cells were stained blue in the Caco-2 monoculture (Figure 1a). In contrast, HT29-MTX cells in monoculture built a comprehensive mucus layer (Figure 1b). In the coculture, a rather incomplete mucus layer was formed (Figure 1c).

### 3.3. MTT Assay

**Figure 2:**
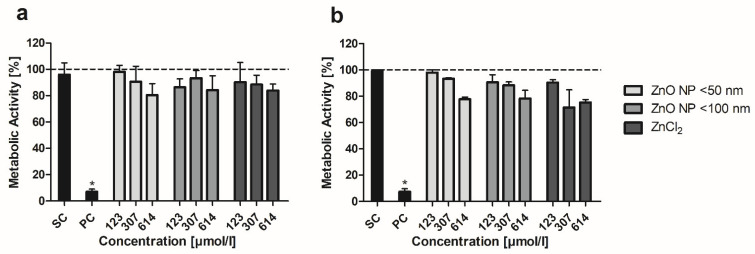
Relative metabolic activity of Caco-2 monoculture (**a**) and Caco-2/HT29-MTX coculture cells (**b**) after 24 h treatment with ZnO NP and ZnCl_2_. SC: solvent control (5% Millipore water); PC: positive control (0.1% Triton X-100 + 10 mM EGTA apical, 10 mM EGTA basolateral). Data are normalized to the untreated control (=100%; dashed line) and expressed as mean + standard deviation; *n* = 4 for (**a**), *n* = 3 for (**b**). Significant differences compared to the untreated control (* *p*  ≤  0.05) were obtained by one-way analysis of variance/Ryan–Einot–Gabriel–Welsh post hoc test.

The MTT assay was conducted to examine the influence of ZnO NP on the metabolic activity of mono- and cocultured cells. In the Caco-2 monoculture, there were no significant differences between ZnO NP- or ZnCl_2_-treated cells and the untreated control (Figure 2a). The lowest metabolic activity was found after 24 h of incubation with 614 µmol/L ZnO NP < 50 nm (79.9 ± 10.5%). In the Caco-2/HT29-MTX coculture, there were also no significant differences between untreated and ZnO NP- or ZnCl_2_-treated cells. Treatment with 614 µmol/L ZnO NP led to slightly decreased metabolic activity (residual activity: 77.8 ± 1.5% for ZnO NP < 50 nm; 78.2 ± 6.2% for ZnO NP < 100 nm; Figure 2b). ZnCl_2_ showed the strongest effects on cocultured cells: metabolic activity decreased to 71.4 ± 13.4% after treatment with 307 µmol/L and 75.2 ± 2.3% after treatment with 614 µmol/L.

### 3.4. Barrier Integrity

The influence of ZnO NP on the barrier integrity of Caco-2 monocultured and Caco-2/HT29-MTX cocultured cells was investigated by taking three different approaches. First, the monolayer integrity was quantified using TEER measurements. After differentiation, the monolayer of Caco-2 monocultured cells reached TEER values of 315.8 ± 21.9 Ω∙cm^2^. The monolayer of Caco-2/HT29-MTX cocultured cells gained TEER values of 322.2 ± 31.0 Ω∙cm^2^.

**Figure 3:**
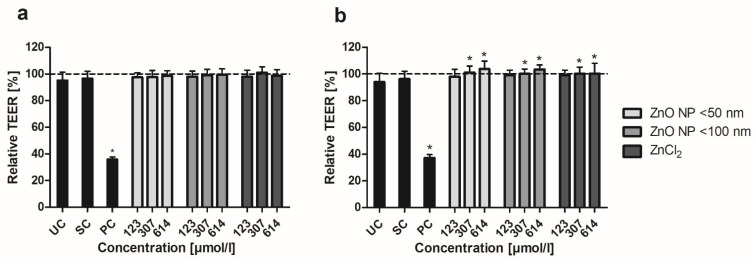
Relative TEER of Caco-2 monoculture (**a**) and Caco-2/HT29-MTX coculture cells (**b**) after 24 h treatment with ZnO NP and ZnCl_2_. UC: untreated control; SC: solvent control (5% Millipore water); PC: positive control (0.1% Triton X-100 + 10 mM EGTA apical, 10 mM EGTA basolateral). Dashed line (=100%) implies TEER before incubation. Data are expressed as mean + standard deviation; *n* = 16. Significant differences compared to UC (* *p*  ≤  0.05) were obtained by one-way analysis of variance/Ryan–Einot–Gabriel–Welsh post hoc test.

To determine the relative TEER values, the ratios of TEER after and before 24 h of incubation with ZnO NP were calculated. In the monoculture, the positive control led to a decreased TEER of 35.7 ± 1.8% (Figure 3a). Treatment with ZnO NP and ZnCl_2_ did not significantly alter the relative TEER values, which were above 97% for all samples. The cocultured cells showed a similarly decreased TEER for the positive control (37.2 ± 2.5%; Figure 3b). In contrast, treatment with ZnO NP and ZnCl_2_ (≥307 µmol/L) led to significantly higher TEER values compared with the untreated control (94.0 ± 6.4% for the untreated control; 100.9 ± 4.9% for 307 µmol/L ZnO NP < 50 nm; 100.0 ± 3.6% for 307 µmol/L ZnO NP < 100 nm; 100.1 ± 4.9% for 307 µmol/L ZnCl_2_).

**Figure 4:**
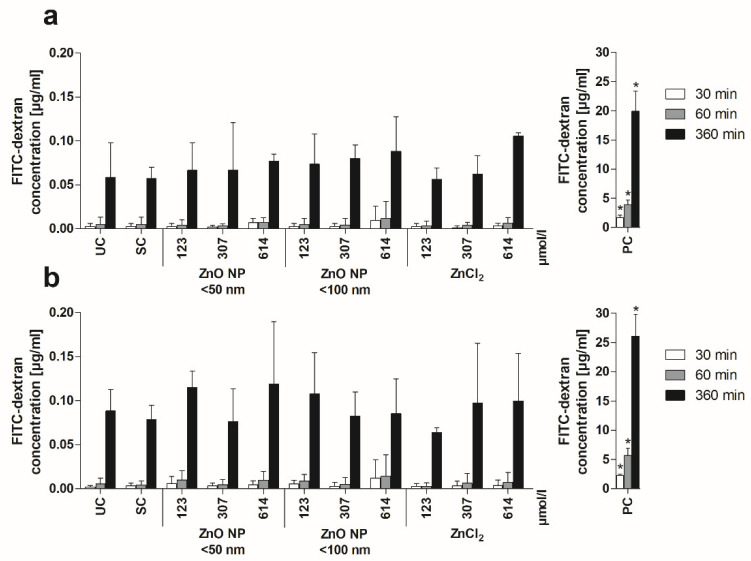
Basolateral FITC-dextran concentrations of Caco-2 monoculture (**a**) and Caco-2/HT29-MTX coculture cells (**b**) after 24 h of treatment with ZnO NP and ZnCl_2_ and subsequent apical addition of FITC-dextran. UC: untreated control; SC: solvent control (5% Millipore water); PC: positive control (0.1% Triton X-100 + 10 mM EGTA apical, 10 mM EGTA basolateral). Data are expressed as mean + standard deviation; *n* = 3. Significant differences compared to UC (* *p*  ≤  0.05) were obtained by one-way analysis of variance/Ryan–Einot–Gabriel–Welsh post hoc test.

Second, monolayer permeability was investigated by measuring the apically added FITC-dextran in the basolateral compartment. Basolateral FITC-dextran concentrations increased over time under both mono- and coculture conditions (Figure 4). Treatment with the positive control resulted in significantly increased basolateral FITC-dextran (after 6 h FITC-dextran treatment: 19.9 ± 3.5 µg/mL for monocultured cells; 26.1 ± 3.7 µg/mL for cocultured cells) compared with the untreated control (0.06 ± 0.04 µg/mL and 0.09 ± 0.02 µg/mL, respectively). In contrast, 24 h of incubation with ZnO NP and ZnCl_2_ did not lead to increased permeability.

**Figure 5:**
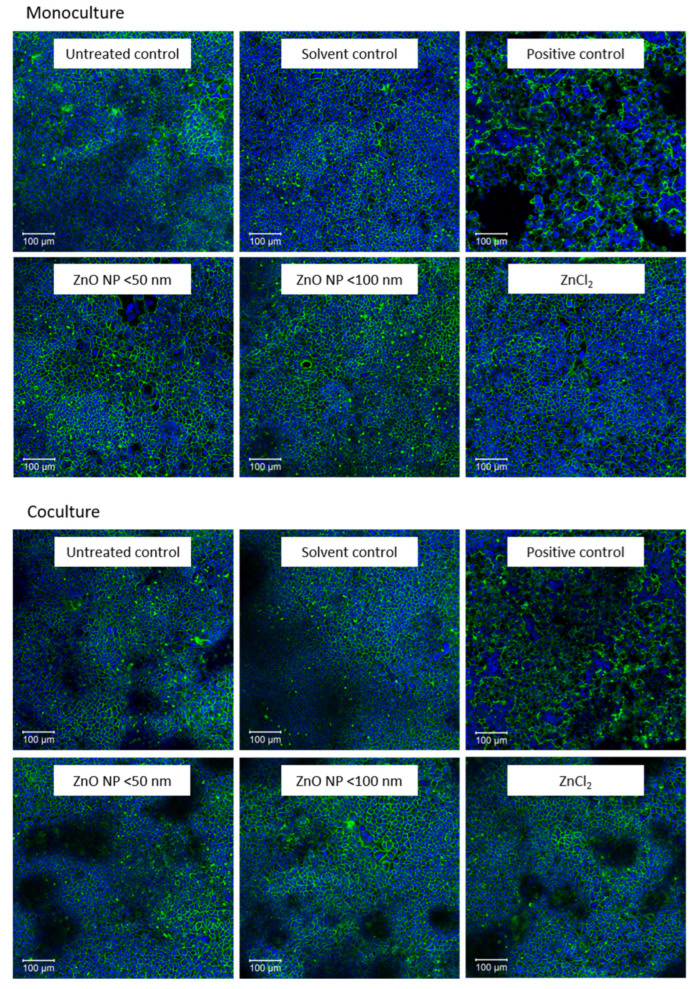
Representative images of fluorescence staining with phalloidin (green) and DAPI (blue) after 24 h incubation with cell culture medium (untreated control), 5% Millipore water (solvent control), 0.1% Triton X-100 + 10 mM EGTA apical, 10 mM EGTA basolateral (positive control), 614 µmol/L ZnO NP (<50 nm or <100 nm) and ZnCl_2_; Caco-2 monocultured cells are shown above and Caco-2/HT29-MTX cocultured cells are shown below.

Third, staining with phalloidin and DAPI should microscopically reveal the influence of ZnO NP on the cytoskeleton and cell nuclei. The effects of the highest concentration (614 µmol/L) of ZnO NP and ZnCl_2_ in comparison with untreated, solvent and positive control cells are shown in Figure 5. Caco-2 monocultured cells formed a flat monolayer, while the cocultured monolayer exhibited elevations. Treatment with the positive control lowered and partially dissolved the cell monolayer anchorage. These cells seemed to be spherical. However, incubation with ZnO NP and ZnCl_2_ had neither influence on cytoskeletal nor nuclear morphology.

### 3.5. Zinc Amount Quantification

**Figure 6:**
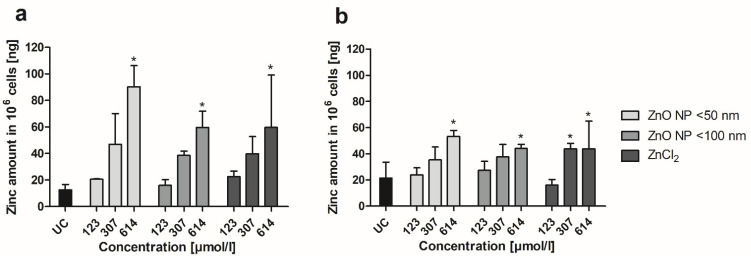
Zinc amount of Caco-2 monoculture (**a**) and Caco-2/HT29-MTX coculture cells (**b**) after 24 h of treatment with ZnO NP and ZnCl_2_. UC: untreated control. Data are expressed as mean + standard deviation; *n* = 3. Significant differences compared to UC (* *p*  ≤  0.05) were obtained by one-way analysis of variance/Ryan–Einot–Gabriel–Welsh post hoc test.

By using ICP-MS, zinc uptake of Caco-2 monocultured and Caco-2/HT29-MTX cocultured cells was determined after ZnO NP and ZnCl_2_ incubation. At first, the cellular zinc amount was measured and calculated for 1 × 10^6^ cells (Figure 6). This revealed a concentration-dependent accumulation of zinc in both cultures, with a smaller increase under coculture conditions. Treatment with 614 µmol/L ZnO NP led to significantly increased zinc amounts in monocultured cells (90.3 ± 15.9 ng zinc in 10^6^ cells for ZnO NP < 50 nm; 59.6 ± 12.2 ng zinc in 10^6^ cells for ZnO NP < 100 nm) and cocultured cells (53.2 ± 4.6 ng zinc in 10^6^ cells for ZnO NP < 50 nm; 44.1 ± 3.1 ng zinc in 10^6^ cells for ZnO NP < 100 nm) compared with untreated controls (monoculture: 12.7 ± 3.9 ng zinc in 10^6^ cells; coculture: 21.5 ± 12.1 ng zinc in 10^6^ cells). Similar effects were observed for ZnCl_2_. There was a significantly increased zinc amount in monocultured cells after treatment with 614 µmol/L ZnCl_2_ (59.8 ± 39.4 ng zinc in 10^6^ cells) and in cocultured cells after treatment with ≥307 µmol/L ZnCl_2_ (43.8 ± 4.2 ng zinc in 10^6^ cells for 307 µmol/L; 43.8 ± 21.2 ng zinc in 10^6^ cells for 614 µmol/L) compared with untreated controls.

**Figure 7:**
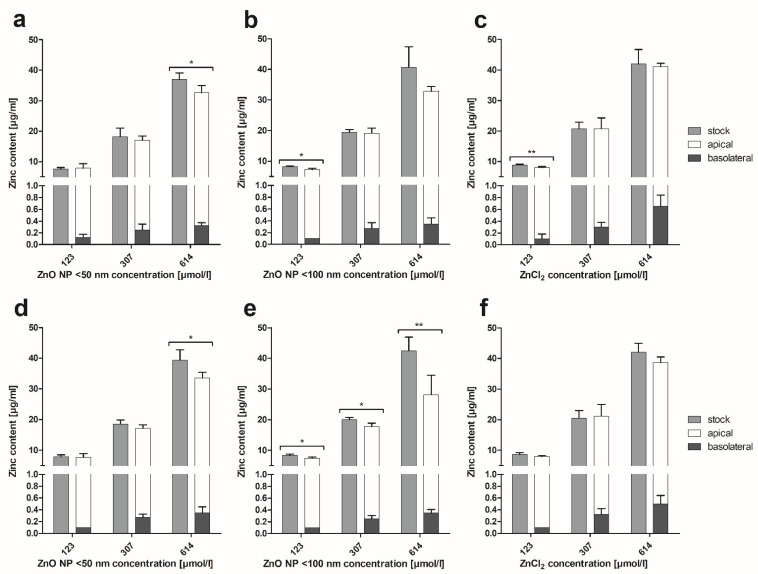
Zinc content of apical and basolateral supernatants of Caco-2 monoculture (**a**–**c**) and Caco-2/HT29-MTX coculture cells (**d**–**f**) after 24 h of treatment with ZnO NP and ZnCl_2_ compared to the stock dispersions. Data are expressed as mean + standard deviation; *n* = 4. Significant differences between zinc amounts before (stock) and after incubation (apical + basolateral; * *p*  ≤  0.05; ** *p*  ≤  0.01) were obtained by unpaired *t*-tests.

In a second approach, zinc concentrations in the apical and basolateral compartments were measured before and after a 24-h treatment with ZnO NP considering mono- and coculture conditions (Figure 7). The presented values were corrected for the basal zinc content of the cell culture medium. After 24 h of incubation, the highest zinc amount was measurable in the apical compartment. Only small amounts of zinc reached the basolateral area (0.07–0.1 µg/mL after treatment with 123 µmol/L ZnO NP or ZnCl_2_; 0.2–0.3 µg/mL after treatment with 307 µmol/L ZnO NP or ZnCl_2_; 0.3–0.6 µg/mL after treatment with 614 µmol/L ZnO NP or ZnCl_2_). For both ZnO NP and ZnCl_2_, the total zinc content was reduced after incubation at all concentrations, reflecting cellular uptake.

## 4. Discussion

ZnO NP offer beneficial properties applicable to the food sector. They can be used for food packaging or for food fortification and as a dietary source of the trace element zinc, which is important for the immune system, cell functions, enzyme activities and signaling in the human body [40,41]. The broad range of applications of ZnO NP will inevitably lead to oral uptake by humans. It is already known that zinc is predominantly absorbed in the small intestine [42], but there is scarce knowledge about the uptake and fate of ZnO NP in the human gastrointestinal tract. Therefore, the aim of this study was to examine the uptake, permeation and effects of two different sizes of ZnO NP on human intestinal cells. To this end, differentiated Caco-2 cells were used in a monoculture and in a coculture with the mucus-producing HT29-MTX cells in a Transwell system.

A characterization of the NP used is an essential prerequisite to ensure the comparability of research results [29]. Brownian motion can be measured by dynamic light scattering, allowing the size distribution of particles in solution, e.g., in cell culture medium, to be determined. Usually, the detected hydrodynamic diameter of NP in a fluid is greater than the primary particle size [43]. Both used ZnO NP exhibited larger hydrodynamic diameters in cell culture medium than the provided primary particle size was (< 50 nm primary particle size versus 162.5 nm mean hydrodynamic diameter; < 100 nm primary particle size versus 219.4 nm mean hydrodynamic diameter). The polydispersity index specifies the broadness of the size distribution. That index was 0.3 for both ZnO NP, which stands for low aggregate and agglomerate formation [44]. The zeta potential indicates the electrostatic charge and thus the charge repulsion/attraction between particles, which affects the stability. The zeta potential for both used ZnO NP was approximately −9 mV. This confirms earlier statements that ZnO NP in cell culture medium carry a predominantly negative charge [45,46,47]. A large zeta potential with values above +25 mV or below −25 mV indicates good physical stability with a sufficient repulsive force. Values within this range can result in particle aggregation due to van der Waals interparticle attraction [48]. These aggregation and agglomeration behaviors have already been observed for ZnO NP [49,50,51,52].

The MTT assay was used to evaluate the cytotoxic potential of ZnO NP [53]. Treatment with ZnO NP had only a weak influence on the metabolic activity of differentiated Caco-2 cells in monoculture and in coculture with HT29-MTX cells. The residual metabolic activity was >75% for all tested concentrations. The salt control ZnCl_2_ showed similar effects on both culture models. In a previous study, we were able to show that undifferentiated Caco-2 cells obviously respond more sensitively to ZnO NP with residual metabolic activity of about 25% for ZnO NP < 50 nm and 36% for ZnO NP < 100 nm after a 24-h treatment with 614 µmol/L ZnO NP [30]. To date, there are only a few studies available using differentiated Caco-2 cells. Sohal and DeLoid [32] showed a dose-dependent reduced metabolic activity of C2BBe1 (subclone of Caco-2)/HT29-MTX coculture cells after a 24-h treatment with 62–500 µmol/L ZnO NP (mean hydrodynamic diameter 244 nm after 48 h in cell culture medium) with a minimum residual activity of about 30%. However, no reduced metabolic activity was observed in C2BBe1 monoculture cells with residual activities above 70%. In contrast to our study, cells were differentiated in 96-well plates without Transwell inserts and, therefore, without separation in apical and basolateral compartments. In addition, treatment of C2BBe1/HT29-MTX coculture cells with other NP (TiO_2_, SiO_2_) also resulted in lower metabolic activity compared with C2BBe1 monoculture cells, which could be due to an unstable coculture. In a study by Mortensen, Moreno and Caffaro [54], 614 µmol/L ZnO NP (hydrodynamic diameter of 300 nm in cell culture medium) did not alter the metabolic activity of differentiated Caco-2 cells. This confirms the assumption that ZnO NP have no cytotoxic effects against an intact intestinal barrier.

In the next step, we examined the influence of ZnO NP on the barrier integrity of Caco-2 monoculture and Caco-2/HT29-MTX coculture cells using different assays. TEER values of differentiated Caco-2 monoculture cells were not altered after 24 h of incubation with ZnO NP but increased significantly in the coculture model. Shao and Wolf [55] showed that zinc stimulates cell differentiation and zonula occludens-1 formation, which leads to an improved intestinal epithelial barrier function. The mucus layer seems to play a key role in zinc availability for the human intestinal mucosa [42], which could explain the improved TEER values of the coculture compared to the monoculture.

Due to the use of the Transwell system, the basolateral FITC-dextran concentrations could be measured after apical addition. Our results revealed no changes in the paracellular permeability after ZnO NP treatment. In addition, fluorescence staining of actin filaments with phalloidin and of cell nuclei with DAPI confirmed the intact monolayer. There were no visible changes in cell morphology due to ZnO NP exposure at concentrations up to 614 µmol/L. The results were similar for ZnCl_2_.

A few studies also examined the barrier integrity of differentiated Caco-2 cells after ZnO NP incubation. Sohal and DeLoid [32] found heterogeneous effects of ZnO NP on the monolayer integrity of their intestinal models. TEER values of C2BBe1 monoculture cells were significantly reduced by up to 50%, while the C2BBe1/HT29-MTX/Raji B triculture barrier was not affected after 24 h of exposure to 250 µmol/L ZnO NP. However, microscopic analyses revealed abnormalities in microvilli. There were gaps in the microvilli on cell surfaces, while the remaining microvilli seemed to be lying on their sides. Tight junction integrity and tight junction protein expression were investigated by staining for zonula occludens-1. No altered expression could be detected in the mono- and triculture after ZnO NP treatment. In a study by Mortensen, Moreno and Caffaro [54], there were also no changes in zonula occludens-1 formation in the differentiated Caco-2 monolayer after 24 h of exposure to 614 µmol/L ZnO NP (mean hydrodynamic diameter 299 nm in cell culture medium). In contrast, the monolayer permeability was significantly increased by AF488-dextran. However, there was no explanation for this effect. In a study by Colombo and Cortinovis [56], apical exposure of differentiated Caco-2 cells with up to 1229 µmol/L ZnO NP did not significantly alter TEER values. Interestingly, basolateral treatment with 12–1229 µmol/L ZnO NP (mean hydrodynamic diameter 953 nm) decreased TEER significantly at all concentrations. Basolateral treatment with smaller ZnO NP (mean hydrodynamic diameter 603 nm) led to decreased TEER values only at the highest concentration (1229 µmol/L). This phenomenon could be due to cell polarity. The basolateral plasma membrane contains a different protein and lipid composition than the apical membrane, which could lead to a different biochemical response to ZnO NP [57].

Finally, cellular zinc uptake and zinc permeation through the monolayer were investigated. While treatment with 614 µmol/L ZnO NP < 50 nm led to a 7-fold higher zinc amount in Caco-2 monoculture cells compared with the untreated control, there was only a 2.5-fold increased zinc amount detectable in coculture cells after ZnO NP exposure. Nevertheless, treatment with ZnO NP < 50 nm resulted in higher cellular zinc contents than the zinc ion control ZnCl_2_, which indicates an NP effect. In both cultures, only very small zinc amounts (0.07–0.65 µg/mL) permeated into the basolateral area, reflecting an intact monolayer barrier. Chang and Choi [58] also investigated ZnO NP (123 µmol/L) permeation through the monolayer of differentiated Caco-2 cells. The basolateral ZnO NP amount increased over a 4 h measurement time, and the smallest ZnO NP used (20 nm primary particle size) had the highest permeation rate compared with larger ZnO NP (90–200 nm and 1–5 µm). Remarkably, cells were treated with ZnO NP suspended in PBS without cell culture medium, and the basolateral area was filled with Hank’s balanced salt solution. The basolateral samples were then analyzed spectrophotometrically. The fluid in which ZnO NP are dispersed has an immense influence on their behavior. Biomolecules interact with NP and build a corona, which modifies the interactions between NP and specific cellular receptors and internalization pathways [59]. Therefore, a direct comparison of our results with those of Chang and Choi [58] is difficult.

Cellular uptake of the trace element zinc is influenced by the amount of zinc in the food, the food composition and the intestinal mucus layer [60,61]. Mucus can bind zinc ions and prevent precipitation at intestinal pH, which should increase the solubility and availability of zinc to the intestinal epithelium [42]. This indicates the potential for higher zinc uptake in a Caco-2/HT29-MTX coculture than in Caco-2 monocultured cells. However, the opposite occurred in our study. The monocultured cells contained much more zinc, but there was very little zinc on the basolateral side in both cultures. In general, protection of the intestinal tissue against foreign substances is an important function of the intestinal mucus [42]. Our results suggest that the mucus formed by HT29-MTX cells kept ZnO NP away from the monolayer underneath.

However, orally ingested ZnO NP are exposed to pH shifts, different enzymes and fluids in the human body, which could influence their physicochemical properties and thus affect cellular uptake and toxicological behavior [62,63]. In the next step, an in vitro digestion could be performed to reach an even better approximation of the physiological circumstances. In addition, the coculture model could be expanded to include M cells, such as Raji B, thus achieving an even better approximation to the in vivo situation.

## 5. Conclusions

Our data indicate that especially differentiated Caco-2 monocultured cells internalize zinc, but this is not reflected in changes in their permeability, metabolic activity and morphology. TEER is positively influenced by both ZnO NP (<50 and <100 nm), particularly in Caco-2/HT29-MTX coculture cells. Only small amounts of zinc (0.07–0.65 µg/mL) permeate the intact monolayer, which means that only small ZnO NP amounts could enter the circulation. Altogether, our results reveal that the cells of an intact intestinal barrier interact with ZnO NP in realistic concentrations (123–614 µmol/L) but do not suffer serious damage.

## Figures and Tables

**Table 1:** Physicochemical properties of zinc oxide nanoparticle (ZnO NP) dispersions.

ZnO NP	Hydrodynamic Diameter (nm)	Polydispersity Index	Zeta Potential (mV)
<50 nm	162.5 ± 12.0	0.3 ± 0.1	−9.1 ± 1.9
<100 nm	219.4 ± 11.3	0.3 ± 0.1	−9.0 ± 2.4

Mean hydrodynamic diameter from dynamic light scattering intensity distribution (zeta average); the measurements were performed with 614 µmol/L ZnO NP dispersions in cell culture medium after ultrasonication.

## Data Availability

The data presented in this study are available on request from the corresponding author.
